# Ultrasound Identification of an Atypical Course of the Posterior Intercostal Artery During Paravertebral Block

**DOI:** 10.7759/cureus.78276

**Published:** 2025-01-31

**Authors:** David Y Chee, Oriana Ng

**Affiliations:** 1 Anesthesiology and Critical Care, Singapore General Hospital, Singapore, SGP

**Keywords:** posterior intercostal artery, regional anesthesia, thoracic paravertebral block, ultrasound-guided, vascular injury

## Abstract

The thoracic paravertebral block (TPVB), while relatively safe, can be associated with significant complications, including inadvertent vascular injury. We describe an ultrasound-guided TPVB where a pulsatile artery was identified between the two transverse processes and in close proximity to the T7-8 paravertebral space, likely the dorsal branch of the posterior intercostal artery. A similar artery was also noted one intercostal space cephalad and caudal of this area. The use of ultrasound allowed for real-time visualization of the needle, minimizing the risk of arterial puncture.

## Introduction

The thoracic paravertebral block (TPVB) is a regional anesthetic technique that is often performed as part of a peri-operative, opioid-sparing, multi-modal analgesia plan. It can be utilized across a wide range of surgeries, including cardiothoracic, breast, and abdominal surgeries. Compared to opioids, TPVB has been shown to provide superior analgesia, reduce post-operative nausea and vomiting, and preserve post-operative pulmonary function, particularly in thoracic surgery [1]. Additionally, when compared to a thoracic epidural analgesia technique, TPVB has been shown to provide comparable pain relief while reducing the risks of complications such as hypotension from bilateral sympatholysis, urinary retention, and pruritus [2].

While TPVBs are relatively easy to perform and have a high success rate, their use is not without risk. Complications specific to the procedure include vascular puncture, pleural puncture, pneumothorax, hemothorax, and dural puncture. Although the overall risk of such complications remains relatively low, they can lead to significant morbidity for the patient [3]. In recent years, there has been a shift towards the use of ultrasound-guided techniques for regional anesthesia. This provides the benefit of real-time visualization of the needle, potentially increasing the success rate of the block while reducing the risk of complications [4].

We describe a case of an ultrasound-guided TPVB where an artery, likely the dorsal branch of the posterior intercostal artery (PIA), was identified within the anticipated needle trajectory. The PIA usually lies deep to the superior costotransverse ligament (SCTL), within the paravertebral space [5]. However, the location of this artery was superficial to the SCTL, at the level of the dorsal surface of the transverse processes. This artery could potentially have been punctured if not for the use of ultrasound. To our knowledge, this is the first time the presence of an artery at this location has been described and visualized on ultrasound. This case highlights the importance and potential benefit of adopting an ultrasound-guided approach to the TPVB.

## Case presentation

A 61-year-old male (height of 170 cm, weight of 53.5 kg) was scheduled for an elective right video-assisted thoracic surgery with a right lower lobectomy. He was otherwise healthy with no past medical history apart from a right lower lobe adenocarcinoma which was an incidental finding on a chest X-ray. Pre-operative examination and investigations were all normal with no evidence of distant metastasis.

**Figure 1:**
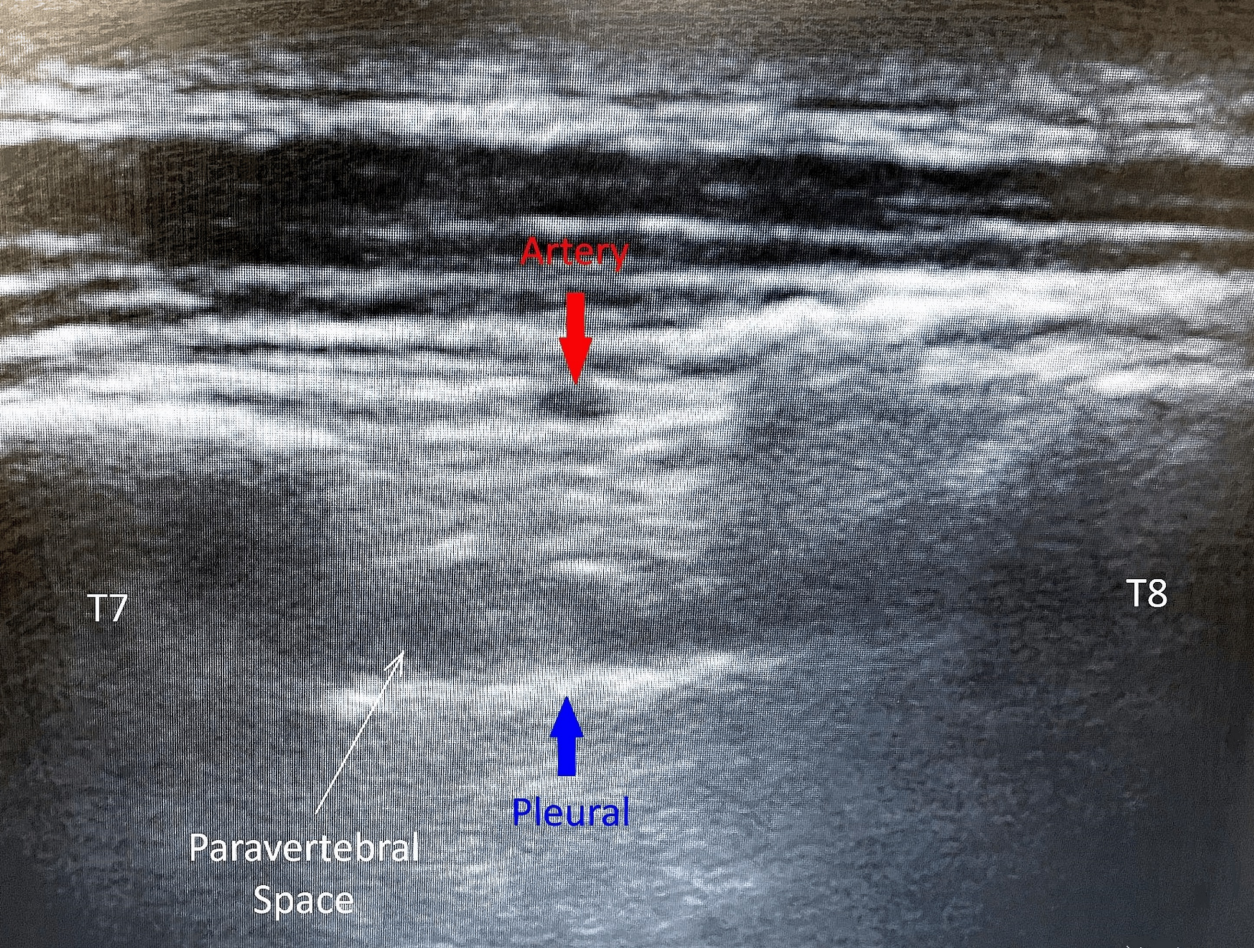
Dorsal branch of the posterior intercostal artery. Location of the dorsal branch of the posterior intercostal artery between the two transverse processes of T7 and T8. The anechoic, circular artery marked by the red arrow is likely the dorsal branch of the posterior intercostal artery, which was superficial to both the paravertebral space and superior costotransverse ligament in this patient.

Post-induction, the patient was placed in the left lateral decubitus position, and an ultrasound-guided right TPVB was planned using the parasagittal out-of-plane approach, as is the usual practice in our department. Using the inferior angle of the scapula as a landmark, a high-frequency linear array transducer was placed approximately 2.5 cm lateral to the midline, and the SCTL at the T7-8 paravertebral space was identified. Prior to needle insertion, a pulsatile, anechoic, circular structure was identified between the two transverse processes of T7 and T8, at the level of their dorsal surfaces, corresponding to a depth of approximately 1.3 cm (Figure 1). On color Doppler imaging, the structure displayed a predominantly red signal with a pulsatile flow pattern. On scanning laterally, the artery appeared to travel in close proximity to the transverse process cranial to it, while medially, it continued to travel in the middle of the intercostal space. This was likely the dorsal branch of the PIA, and a similar pulsatile artery was also noted one level above and below the intended paravertebral space.

Thus, care was taken to ensure that the needle puncture and trajectory remained caudal to the artery at all times in order to minimize the risk of arterial puncture. Real-time ultrasound visualization of the needle, as well as hydrodissection, was used to place the needle in the intended paravertebral space. After confirming negative aspiration, the block was performed uneventfully at the T4-5 and T7-8 paravertebral spaces.

The TPVB worked well and good peri-operative pain control was achieved. No evidence of bleeding was noted at the paravertebral region during thoracoscopic surgery and the patient had an uneventful recovery. A note was made in his anesthetic record detailing the findings during his TPVB procedure, and the use of ultrasound guidance was recommended for future TPVB, should he require a repeat procedure.

## Discussion

The TPVB provides excellent peri-operative analgesia and is often used for cardiothoracic and breast surgeries. Overall, the risk of complications from a TPVB appears relatively low, with most studies citing an incidence of around 5% or less [3]. In particular, the risk of vascular puncture was reported as 3.8% in a prospective study [6]. While most of these cases remain asymptomatic with no adverse sequelae, there have been case reports of significant paravertebral hematoma and even pulmonary hemorrhage following TPVB, particularly when performed using a landmark approach [5,7].

The thoracic paravertebral space contains multiple key structures that are at risk of inadvertent injury during a TPVB. These include the intercostal artery and vein, intercostal spinal nerve, and sympathetic trunk [8]. While most of these vessels lie close to or within the thoracic paravertebral space itself, this study demonstrates that anatomical variations of the dorsal branch of the PIA may run more superficially, close to the dorsal aspect of the transverse processes. This may put it at risk of inadvertent puncture during TPVB, particularly with the landmark technique as the needle is usually walked off either cranially or caudally after hitting the transverse process.

Conventionally, the PIA has been thought to travel within the subcostal groove, in close relation to the rib above it [8]. However, a study looking at the course of the posterior intercostal artery using computed tomography angiography found it to have significant variability and tortuosity, particularly at the level of the posterior paravertebral space [9]. In addition, at the posterior paravertebral space from the sixth to eighth intercostal space, the PIA tends to lie more towards the middle of the intercostal space. This appears to be in keeping with our findings during this patient's TPVB.

This study highlights the potential benefits of ultrasound guidance when performing a TPVB block, and serves as a reminder for practitioners to carefully identify any vascular structures in the needle trajectory prior to performing the block. The use of an ultrasound-guided technique may also achieve a higher success rate of surgical anesthesia and post-operative analgesia [10]. Color Doppler has also been advocated to assist in the identification of blood vessels prior to performing a TPVB.

Several alternatives to the TPVB have also been described, such as the erector spinae plane (ESP) and mid-point transverse process to pleura (MTP) blocks. The current evidence comparing the ESP, MTP, and TPVB primarily stems from observational studies or small randomized controlled trials (RCTs), resulting in the quality of evidence that is generally low [11-13]. A recent meta-analysis of RCTs suggests that the TPVB provides superior analgesic efficacy and pain relief compared to the ESP block [14]. However, there was significant heterogeneity, low quality of evidence, and the observed difference in pain scores was small, which may not be clinically significant. One of the purported advantages of these alternative blocks is the lower risk of vascular injury, given that the targets are more superficial and the needle does not traverse the SCTL into the paravertebral space [15,16]. However, this study highlights that there are still important vascular structures superficial to the SCTL as the dorsal branch of the PIA may travel in close proximity to the needle trajectory of these alternative blocks. Hence, careful identification of vascular structures with ultrasound should still be undertaken prior to performing these procedures.

## Conclusions

This study highlights the potential risks posed by anatomical variations of the PIA, specifically the superficial course of its dorsal branch. This increases the likelihood of vascular injury during a TPVB, particularly when a landmark approach is used. This underscores the essential role of real-time ultrasound guidance in identifying potential vessels within the needle trajectory, allowing for precise adjustments and minimizing the risk of complications. Careful pre-procedure scanning and color Doppler are invaluable in identifying such anatomical variations, enhancing the overall safety and efficacy of the TPVB.
